# Changes in the proteomic profile of blood serum in coronary atherosclerosis

**DOI:** 10.2478/jomb-2019-0022

**Published:** 2020-01-23

**Authors:** Ekaterina M. Stakhneva, Irina A. Meshcheryakova, Evgeny A. Demidov, Konstantin V. Starostin, Sergey E. Peltek, Michael I. Voevoda, Yuliya I. Ragino

**Affiliations:** 1 Siberian Branch of Russian Academy of Sciences, Institute of Internal and Preventive Medicine - A branch of Institute of Cytology and Genetics, Novosibirsk, Russia; 2 Siberian Branch of Russian Academy of Sciences, Institute of Cytology and Genetics, Novosibirsk, Russia

**Keywords:** proteomics, mass spectrometry, two-dimensional electrophoresis, atherosclerosis, ceruloplasmin, ceruloplazmin, ateroskleroza, dvodimenzionalna elektroforeza, masena spektrometrija, proteomika

## Abstract

**Background:**

Our aim was to study changes in the serum proteomic profile in coronary atherosclerosis.

**Methods:**

The study involved two groups of patients: 1) men with coronary heart disease and coronary atherosclerosis (n = 15); 2) control (n = 15): men without coronary heart disease. The object of this study was blood serum. Separation of proteins for the investigation of differences in serum protein components was performed by two-dimensional electrophoresis. Identification of protein fractions was carried out using peptide mass maps by the matrix-assisted laser desorption ionization method.

**Results:**

In blood serum samples from patients with coronary atherosclerosis, protein separation in two-dimensional gels with mass-spectrometric identification revealed an increase of some proteins: hemopexin, transthyretin (monomeric form), retinol-binding protein 4, and components of the complement system: C3 (chain B) and C9. There was a decrease of some proteins: kininogen, zinc finger protein 133, and B-cell CLL/lymphoma 6 member B protein. Comparisons between the experimental and control group were carried out in protein fractions where the protein amount differed more than 1.5-fold (p < 0.05).

**Conclusions:**

Proteome profiling of serum revealed a change in the content of kininogen, hemopexin, transthyretin, retinol-binding protein, and proteins of the complement system (C9, and C3) in coronary atherosclerosis. The contribution to the differential expression of a protein was often made by isoforms of the protein, particularly transthyretin. The change in the concentrations of functionally interacting proteins, such as transthyretin and retinol-binding protein, were noted.

## Abstract (sr)

**Uvod:**

Cilj ove studije bio je proučavanje serumskog proteomičkog profila kod koronarne ateroskleroze.

**Metode:**

U istraživanju su učestvovale dve grupe pacijenata: 1) muškarci sa koronarnom bolešću srca i koronarnom aterosklerozom (n = 15); 2) kontrolna grupa (n = 15): muškarci bez koronarne bolesti srca. Predmet studije bio je krvni serum. Odvajanje proteina za ispitivanje razlika u komponentama proteina u serumu izvršeno je dvodimenzionalnom elektroforezom. Identifikacija frakcija proteina izvršena je pomoću mapa masa peptida metodom matricom potpomognute laserske desorpcione jonizacije.

**Rezultati:**

U uzorcima krvnog seruma pacijenata sa koronarnom aterosklerozom, razdvajanjem proteina u dvodimenzionalnim gelovima masenom spektrometrijskom identifikacijom utvrđeno je povećanje nekih proteina: hemopeksin, transtiretin (monomerni oblik), retinol-vezujući protein 4 i komponente sistema komplementa: C3 (lanac B) i C9. Došlo je do smanjenja nekih proteina: kininogena, proteina cinkovog prsta 133, i B-ćelijskog CLL / limfoma 6 člana familije B proteina. Poređenja između eksperimentalne i kontrolne grupe vršena su u proteinskim frakcijama gde se količina proteina razlikovala više od 1,5 puta (p < 0,05).

**Zaključak:**

Proteomska profilacija seruma otkrila nam je promenu u sadržaju kininogena, hemopeksina, transtiretina, retinol-vezujućeg proteina i proteina komplementarnog sistema (C9 i C3) kod koronarne ateroskleroze. Doprinos diferencijalnoj ekspresiji proteina je često uzrokovan izoformima proteina, naročito transtiretinom. Zabeležena je promena koncentracija funkcionalno intereagujućih proteina, kao što je transtiretin i retinol-vezujući protein.

## Introduction

Early diagnosis of atherosclerosis is important for the prevention of cardiovascular diseases (CVDs) and adjustment of relevant therapies. Accumulation of knowledge about the pathogenesis of CVD together with the development of modern research methods in recent decades has intensified the search for proteins that are potential prognostic and diagnostic biomarkers of CVD. Even though the number of studied candidate proteins is continually growing, their role in the pathogenesis of coronary atherosclerosis is not always entirely clear. Understanding the changes in blood proteins in early atherosclerosis will help identify new candidate biomarkers for the detection of this disease before the onset of clinical manifestations. The proteomic analysis allows researchers to overcome the difficulties associated with a wide concentration range of proteins in the blood and their posttranslational modifications. For early diagnosis of the disease, special attention is paid to acute phase proteins and proteins involved in the implementation of an immune response. Proteins of the complement system, which is the most important factor of microbial resistance of the human body, take part in immune response [1]. Many studies show activation of the complement system in atherosclerosis [1] [2] [3]. Ceruloplasmin is a specific plasma glycoprotein that belongs to the family of acute phase proteins, has pro- and anti-inflammatory properties, and therefore its involvement in atherosclerosis is a controversial topic. In patients with coronary heart disease (CHD), there is a decrease in the level of ceruloplasmin [4]. Some studies associate high levels of ceruloplasmin with heart failure [5]. In our recent study, we demonstrated decreased ceruloplasmin levels in the blood serum of patients with coronary atherosclerosis. However, the level of complement component C4 increased in these patients [6].

This study aimed to evaluate changes in blood serum proteins in patients with CHD and angiographically verified coronary atherosclerosis as compared with a control group.

## Materials and Methods

The study included 30 people: an experimental group of 15 men with CHD and angiographically verified coronary atherosclerosis. The age of the patients was 49.8 ± 1.0 (mean ± SD). The control group consisted of 15 men without CHD, according to clinical and functional studies. The age in the control group was 52.2 ± 0.7. All the subjects signed an informed consent form to participate in the study.

All the patients had their blood drawn from the ulnar vein on an empty stomach in the morning. For this experiment, a mixture of blood serum samples from patients with coronary atherosclerosis was compared with a mixture of serum samples from the control group.

To isolate and purify the samples, the Aurum Serum Protein Mini Kit (Bio-Rad) was employed to remove up to 90% of serum albumin and immunoglobulins from the samples.

Whey protein separation was performed by twodimensional electrophoresis (2DE). The protein was buffered (7 mol/L urea, 2 mol/L thiourea, 4% 3-[(3cholamidopropyl)-dimethylammonium)-1-propanesulfonate (CHAPS), 65 mmol/L dithiothreitol (DTT), 0.4% ampholytes pH 3-10) and the protein concentration was determined in the Quick Start™ Bradford Protein Assay (Bio-Rad) with bovine g-globulin as a standard.

For analytical purposes, for two-dimensional sodium dodecyl sulfate (SDS) polyacrylamide gel electrophoresis, each gel was loaded with 100 μg of protein from experimental or control samples; for preparative electrophoresis, 320 μg of protein was loaded per gel. Separation in the first direction of 2DE was carried out in the chamber PROTEAN IEF Cell (Bio-Rad) in 17 cm strips, pH range 4-7 (Ready-Strip™ IPG Strip, Bio-Rad, USA). The strips were rehydrated and loaded with 300 μL of the samples; separation lasted for 12 hours at a current of 50 mA per strip and at 20 °C; isoelectrofocusing was carried out at the following settings: 250 V for 1 h, 1000 V for 1 h, then the voltage was linearly increased to 10,000 V for 3 h, and isoelectrofocusing of the sample was performed until the total number of volt-hours reached 60,000. Prior to separation in the second direction, the strips were stored at -80 °C, then equilibrated in a buffer (6 mol/L urea, 2% SDS, 20% glycerol, 0.375 mol/L Tris-HCl pH 8.8) supplemented with 130 mmol/L DTT for 15 min and then for 15 min in the buffer supplemented with 200 mmol/L iodo cyanide, overlaid on a 12% polyacrylamide gel with SDS (acrylamide/bis acrylamide in the ratio of 37.5:1, 0.1% of SDS, 0.375 mmol/L Tris-HCl pH 8.8, 0.05% tetramethylethylenediamine (TEMED), 0.05% of ammonium persulfate). Introduction of proteins into the gel was carried out in the electrophoretic chamber PROTEAN® II xi 2-D Cell (Bio-Rad) at a current of 16 mA, followed by separation of the proteins at a current of 30 mA.

The gels were stained with fluorescent dye Sypro Ruby (Bio-Rad Laboratories). The coloured gels were visualized by means of a VersaDoc MP4000 gel documentation system (Bio-Rad).

Gel images were analyzed in PDQuest Advanced-8.0.1 software (Bio-Rad Laboratories). The analysis involved three technical repeats of the experimental and control samples (Student's *t*-test at the p < 0.05 level of significance).

The protein fractions were cut out of the gels using the EXQuest Spot Cutter (Bio-Rad), and the gel pieces were washed in distilled water and incubated in a 50 mmol/L solution of ammonium bicarbonate in 50% acetonitrile for 20 min at room temperature. Next, the gel pieces were dried with acetonitrile, and tryptic hydrolysis of the proteins was carried out by rehydration of the gel with a solution of modified pork trypsin (Trypsin Gold, Mass Spectrometry Grade, Promega, USA) at a concentration of 15 μg/mL in 50 mmol/L ammonium bicarbonate. Hydrolysis was allowed to proceed for 12 hours at 37 °C.

Mass-spectrometric identification of the obtained peptides of each fraction was carried out on an Ultraflex TOF TOF (Bruker Daltonics) instrument by the matrix-assisted laser desorption ionization (MALDI) method (with searching of the NCBI database) using α-cyano-4-hydroxycinnamic acid as a matrix in 70% acetonitrile with 1% of trifluoroacetic acid.

## Results and Discussion

The analysis of the differential expression of proteins involved three technical replicates of each sample. Via comparisons, a group of protein fractions was identified where the protein amounts differed by more than 1.5-fold between the experiment and the control group (p < 0.05). Preparative gels were set up to identify the proteins. Loading and separation of the preparative amount of a sample were possible due to the removal of albumin from the samples.

**Figure 1:**
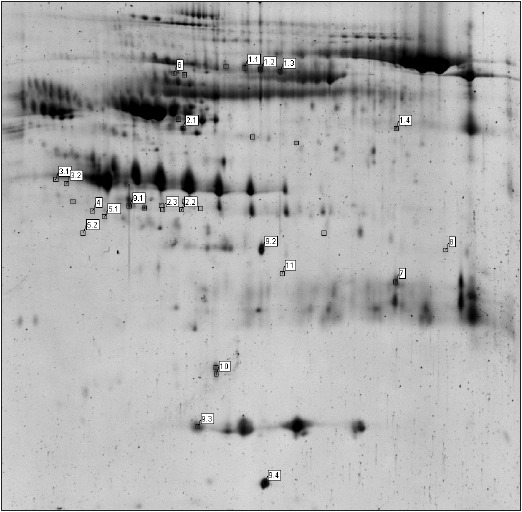
A picture of a representative two-dimensional gel showing separation of an analytical amount of serum proteins Fractions of differentially expressed proteins (p < 0.05 relative to the control) are indicated as squares in the pool of samples from patients with coronary atherosclerosis. The identified proteins are indicated with ID numbers (see Table 1).

A picture of the analytical gel with the identified protein fractions is shown in Figure 1. The results of mass-spectrometric identification are given in Table 1.

**Table 1:** The results of mass-spectrometric identification of differentially expressed proteins Δ is the fold difference in the concentration of proteins in the serum of patients with coronary atherosclerosis relative to the control; “sc” means sequence coverage. In the first column, the ID numbers correspond to the names of electrophoretic spots in Figure 1; ID numbers with a decimal point denote isoforms of a protein

Spot ID No.	NCBI ID	Name of the protein	Mass, Da	pI	Sc, %	Score	Δ
1.1	gi|386789	hemopexin precursor, partial	51512	6.57	40	70	+1.7
1.2	gi|386789	hemopexin precursor, partial	51512	6.57	26	72	+2
1.3	gi|386789	hemopexin precursor, partial	51512	6.57	26	74	+1.6
1.4	gi|386789	hemopexin precursor, partial	51512	6.57	28	78	+5.7
3.1	gi|545478558	zinc finger protein 133 isoform f	70201	9.43	33	70	-11
3.2	gi|545478558	zinc finger protein 133 isoform f	70201	9.43	31	68	-10
4	gi|62898910	kininogen 1 variant	47823	6.29	36	74	-1.8
5.1	gi|78101271	chain C, human complement component, C3c	39463	4.79	58	102	-3.5
5.2	gi|78101271	chain C, human complement component, C3c	39463	4.79	43	79	-2.4
6	gi|2258128	complement 9	61728	5.42	26	91	+2
7	gi|78101270	chain B, human complement component, C3c	21482	5.84	45	114	+2.9
9.1	gi|212374952	chain A, crystal structure of transthyretin, variant	13741	5.35	89	176	+4.6
9.2	gi|377656323	chain A, transthyretin	12869	5.33	81	82	-2.7
9.3	gi|377656323	chain A, transthyretin	12869	5.33	81	83	+3.6
9.4	gi|2098255	chain A, transthyretin	13829	5.35	59	67	+3.7
10	gi|305677614	chain A, RBP4	20018	5.24	77	105	+9

Most of the proteins whose content was different in the serum samples of patients with coronary atherosclerosis belong to the groups of inflammatory proteins and transport proteins: transthyretin, RBP4 chain A, hemopexin, and proteins components of the complement system: C3, and C9. Besides, in the serum samples of the patients, the levels of kininogen and transcription regulators (an isoform of zinc finger protein 133 and B-cell CLL/lymphoma 6 member B protein) were different from those in the control group.

In our study, in the pool of serum samples from patients with coronary atherosclerosis, we observed an increase of the following proteins: hemopexin, transthyretin, RBP4 chain A, proteins of the complement system: C9, and a C3 part (chain B).

The complement system includes ∼20 interacting proteins involved in an immune response. This system is characterized by a fast, repeatedly enhanced response to an antigen signal, and the product of one reaction is the catalyst for the subsequent one. In the absence of a relevant antigen, complement components are in an inactive state. Successive reactions of proteolytic activation of the early compo nents C1, C2, C3, and C4 take part in the development of an inflammatory process. C4b and C2a bind to each other on the surface of a pathogen and form a C3 convertase. Its formation is the main event of the whole reaction cascade. The C3-convertase enzyme splits the C3 component into fragments C3a and C3b. A larger fragment (C3b) binding to C3-convertase, forms C5-convertase, catalyzing the digestion of C5 into fragments C5a and C5b. The released C5b remains fixed on the membrane and sequentially binds C6, C7, C8, and C9, thereby forming a membrane-attacking complex that lyses the target cell by forming a transmembrane channel [1]. It is known that the complement system is activated in atherosclerosis and CVD [2] [3].

This study revealed upregulation of complement components C3 (chain B), and C9 and downregulation of complement component C3 (chain C) in the serum pool of patients with coronary atherosclerosis. Comparison of the positions of these proteins on the gel with their theoretical molecular weight suggests that, apparently, we have found some isoforms C3 (chain C). Nowadays, there are no data on the relation between serum concentrations of various isoforms of complement component C3 chains and atherosclerosis.

**Figure 2:**
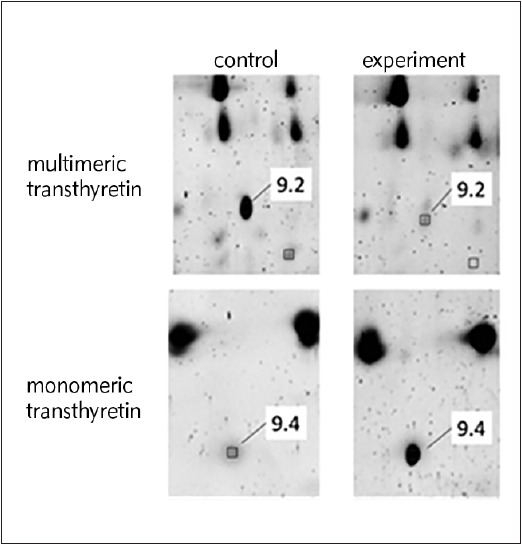
Fragments of two-dimensional gels (with separated proteins from serum samples) containing transthyretin isoforms

Transthyretin and retinol-binding protein are functionally interacting proteins that form a transport complex for vitamin A. In our study, three upregulated isoforms of transthyretin (9.1, 9.3, and 9.4) were identified in the serum of the patients (Table 1), and one isoform (9.2) which was downregulated (Table 1). Summing of the intensities of staining of all the detected isoforms showed that the total concentration of transthyretin was higher in the serum of the patients. Pictures of gel areas containing these isoforms are presented in Figure 2.

Isoforms 9.2 and 9.4 have the same isoelectric point and, apparently, 9.4 is a monomer, whereas 9.2 is a multimeric form; thus, the monomeric form of transthyretin dominates in the serum of patients with atherosclerosis. Transthyretin is synthesized in the liver and is present in plasma in the form of a homotetramer totalling 55 kDa, consisting of subunits with a molecular weight of 13.8 kDa. This protein ensures the transport of thyroxine and retinol. Improper assembly of the tetramer, e.g., due to point mutations, can lead to the formation of amyloid fibrils, which often occurs in the affected arteries [7].

The results of our work are consistent with the findings of Cubedo et al. [8], where an inverse relationship between the concentration of trimeric transthyretin and the risk of CVD was demonstrated for the first time.

Retinol-binding protein (RBP) is a low-molecular-weight protein (21 kDa) of the lipocalin family containing 8 loops of a β-sheet structure specifically binding vitamin A. RBP biosynthesis is carried out in the liver. In blood plasma, RBP binds to transthyretin, forming a complex that functions as a system for the transport of vitamin A. It is reported that there is a correlation between the concentration of this transport complex for retinol (as well as the concentration of free RBP) and thickness of the intima-media, which is diagnostic of atherosclerosis of the carotid artery [9].

RBP4 concentration is a cardiovascular risk factor associated with insulin resistance and CHD; thus, RBP4 can serve as a marker of metabolic complications and atherosclerosis for evaluation of CHD [10]. Also, it is known that in patients with coronary atherosclerosis, elevated levels of RBP4 correlate with disease severity [11].

Hemopexin can bind to hemoglobin and free heme, thereby protecting the body from possible oxidative damage. The concentration of hemopexin increases several fold during inflammation, and it is also referred to as a glycoprotein of the acute phase [12]. It is known that iron accumulates in atherosclerotic plaques and other affected areas of arteries, and in a catalytically active form, can cause proatherogenic events, such as the production of reactive oxygen species and lipid peroxidation [13]. Therefore, heme-binding hemopexin is regarded by many researchers as a protective protein in this process, although its role in atherosclerosis is not fully understood [14]. We revealed the upregulation of four hemopexin isoforms in the serum of patients with coronary atherosclerosis.

Analysis of proteins downregulated in the pool of blood serum from patients with coronary atherosclerosis identified three isoforms of ceruloplasmin. We identified downregulation of one isoform and two fragments of ceruloplasmin in the serum of patients with coronary atherosclerosis.

In addition to acute phase proteins, in blood serum, we noted downregulation of kininogen and isoforms of two negative regulators of transcription (zinc finger protein 133 and B-cell CLL/lymphoma 6 member B protein) in patients with coronary atherosclerosis compared to the control group.

Kininogens are inactive multifunctional glycoproteins, whose molecules consist of a single polypeptide chain; kininogens are synthesized mainly by hepatocytes and are subjected to post-translational glycosylation before secretion into the bloodstream. In human blood plasma, two present kininogens are a low-molecular-weight isoform and a high-molecular-weight one. Kininogens are precursors of bradykinin and kallidin, proteins causing vasodilation and contraction of smooth muscles, respectively. Thus, kininogens participate in inflammation, blood pressure control, blood coagulation, and pain. As to CVDs, the role of kinins is ambiguous: on the one hand, kinins are known for their ability to induce the synthesis of nitric oxide and prostacyclin, mediating cardioprotection, and on the other hand, bradykinin can promote inflammation, fibroplasia, and fibrosis after myocardial infarction in rats [15]. In rats, a genetic deficiency in high-and low-molecular-weight kininogen during consumption of an atherogenic diet does not lead to the development of atherosclerosis but causes aortic aneurysms [16].

The main functions of zinc finger proteins are an interaction (as transcription factors) with DNA or RNA. To date, there is insufficient data in the literature on the functions of zinc finger protein 133, which was found to be differentially expressed in our study, but presumably, it is a transcriptional regulator. According to the study by Stene et al. [17], a singlenucleotide polymorphism in the sequence of another known zinc finger protein which is a lipid metabolism regulator is associated with a risk of atherosclerosis and ischemia. We identified zinc finger protein 133 in two electrophoretic protein spots, which were upregulated in the serum of the patients. Further research is needed to determine the relation of this possible transcription regulator with the pathogenesis of atherosclerosis.

## Conclusion

According to our study, during coronary atherosclerosis, level of proteins kininogen, zinc finger protein 133, and B-cell CLL/lymphoma 6 member B protein are downregulated. In this disease, there is an increase of protein components of the complement system - in particular C3 (chain B), and C9 - and transport proteins transthyretin and retinol-binding protein. The connection of retinol metabolism with CVDs indicates that for the diagnosis of early stages of atherosclerosis, it makes sense to find not only unrelated but also functionally interacting biomarkers. The separation of proteins in two-dimensional gels and subsequent mass-spectrometric identification made it possible to detect protein isoforms. It is shown that in the serum of patients, there is a change in not only the concentration but also the ratio of the isoforms of transthyretin, with a predominance of the monomeric form. It is possible that the differential expression of isoforms of the C and B chains of complement component C3 is associated with atherogenesis.

Suitability of the identified differentially expressed proteins as biomarkers of coronary atherosclerosis requires further studies on their potential role in the development of this disease.

## Additional information

### Ethics approval and consent to participate

The study protocol was approved by the local Ethics Committee of the Institute of Internal and Preventive Medicine (a branch of the Institute of Cytology and Genetics, the Siberian Branch of the Russian Academy of Sciences, Novosibirsk, Russia). Written informed consent to be examined and to participate in the study was obtained from each subject.

### Availability of data and material

The datasets before and after the analysis in this study are available from the corresponding author on reasonable request.

### Funding

This study was conducted within the framework of RFBR project No. 18-415-540006 p_a as part of the budget topic in state assignment No. 0324-2018-0001.

*Acknowledgements*. The English language was corrected and certified by shevchuk-editing.com.

## Consent for publication

All the authors meet the International Committee of Medical Journal Editors (ICMJE) criteria for authorship in this manuscript, take responsibility for the integrity of the work as a whole, and have given final approval to the version to be published.

## Competing interests

The authors declare that they have no conflict of interest associated with the publication of this article.

## Authors’ contributions

Ekaterina M. Stakhneva: analysis and interpretation of the data, drafting of the manuscript, literature review, and participation in discussion

Irina A. Meshcheryakova: experiment planning, sample preparation, 2D electrophoresis, analysis and interpretation of the data, literature review, and participation in discussion

Evgeny A. Demidov: analysis and interpretation of the mass-spectrometric data

Konstantin V. Starostin: analysis and interpretation of the mass-spectrometric data

Sergey E. Peltek: analysis and interpretation ofthe results of 2D electrophoresis and mass-spectrometric data

Michael I. Voevoda: design and critical revisionof the manuscript

Yuliya I. Ragino: study conception and design and critical revision of the manuscript

## Conflict of interest statement

The authors stated that they have no conflicts ofinterest regarding the publication of this article.

## List of abbreviations

2DE, two-dimensional electrophoresis; CHD, coronary heart disease; CVD, cardiovascular disease; DNA, deoxyribonucleic acid; SDS, sodium dodecyl sulfate, RBP, retinol-binding protein; RNA, ribonucleic acid.
